# The Effect of Hypoxia on the Stemness and Differentiation Capacity of PDLC and DPC

**DOI:** 10.1155/2014/890675

**Published:** 2014-02-20

**Authors:** Yinghong Zhou, Wei Fan, Yin Xiao

**Affiliations:** ^1^Institute of Health and Biomedical Innovation, Queensland University of Technology, Brisbane, QLD 4059, Australia; ^2^Australia-China Centre for Tissue Engineering and Regenerative Medicine (ACCTERM), Brisbane, QLD 4059, Australia; ^3^The State Key Laboratory Breeding Base of Basic Science of Stomatology (Hubei-MOST) and Key Laboratory of Oral Biomedicine Ministry of Education, School and Hospital of Stomatology, Wuhan University, Wuhan 430079, China

## Abstract

*Introduction*. Stem cells are regularly cultured under normoxic conditions. However, the physiological oxygen tension in the stem cell niche is known to be as low as 1-2% oxygen, suggesting that hypoxia has a distinct impact on stem cell maintenance. Periodontal ligament cells (PDLCs) and dental pulp cells (DPCs) are attractive candidates in dental tissue regeneration. It is of great interest to know whether hypoxia plays a role in maintaining the stemness and differentiation capacity of PDLCs and DPCs. *Methods*. PDLCs and DPCs were cultured either in normoxia (20% O_2_) or hypoxia (2% O_2_). Cell viability assays were performed and the expressions of pluripotency markers (Oct-4, Sox2, and c-Myc) were detected by qRT-PCR and western blotting. Mineralization, glycosaminoglycan (GAG) deposition, and lipid droplets formation were assessed by Alizarin red S, Safranin O, and Oil red O staining, respectively. *Results*. Hypoxia did not show negative effects on the proliferation of PDLCs and DPCs. The pluripotency markers and differentiation potentials of PDLCs and DPCs significantly increased in response to hypoxic environment. *Conclusions*. Our findings suggest that hypoxia plays an important role in maintaining the stemness and differentiation capacity of PDLCs and DPCs.

## 1. Introduction

The regeneration of hard tissue has always been a challenging issue. Although there is a broad range of treatment options available, such as tissue transplantation [1], growth factor delivery [2], and the application of biomaterials [3, 4], the reconstitution of lost structures is still far from satisfaction. During the past decade, advances in the research of cell-based therapy have offered new insights into dental tissue regeneration [5]. Currently, PDLCs and DPCs have received intensive attention because they both possess the ability to differentiate into multiple cell types, which promote structural and functional repairs for dental tissue engineering [6]. However, despite being promising stem cell reservoir for dental tissue regeneration, PDLCs and DPCs inevitably undergo replicative senescence under current culture conditions, resulting in cellular phenotypic changes [7–9]. Therefore, maintaining the stemness of PDLCs and DPCs becomes very important for clinical application.

Recent studies suggest that stem cells are localized in the microenvironment of low oxygen [10, 11], indicating that hypoxia may be critical for stem cell maintenance. Hypoxia has been shown to regulate several cellular processes and signal transductions via hypoxia inducible factor-1 (HIF-1) [12, 13], which consists of two subunits, HIF-1*α* and HIF-1*β*. Being a hypoxia response factor, HIF-1*α* is regulated by the cellular oxygen (O_2_) concentration and determines the transcriptional activity of HIF-1 [14]. Research on neural and hematopoietic precursors [15, 16] indicates that low O_2_ tension in cell culture has positive effects on the *in vitro* survival and self-renewal of stem cells. Hypoxic microenvironment assists in maintaining the multipotent property of embryonic stem cell (ESC) [17, 18]. On the other hand, recent reports show that differentiated cells can be reprogrammed to a more primitive state by the introduction of Oct-4, Sox2, and c-Myc and the expression of these markers is essential in maintaining the stem cell properties [19, 20]. However, the effects of hypoxia on the expression of these reprogramming markers and stemness maintenance of PDLCs and DPCs are not well illustrated. In this study, we examined the cell vitality, evaluated the expression of pluripotency markers, and assessed the differentiation potential of PDLCs and DPCs under both normoxic and hypoxic culture conditions.

## 2. Materials and Methods

### 2.1. Sample Collection and Cell Culture

Impacted third molars (*n* = 6) were collected from healthy adults (18–30 years old) after obtaining informed consent from each donor and ethics approval from the Ethics Committee of Queensland University of Technology. Periodontal ligament was gently separated from the middle third of the root surface, minced with scalpels, and rinsed with phosphate buffered saline (PBS) [21]. Dental pulp was removed from the root canal, dissected to small pieces, and rinsed with PBS [7]. The tissue explants were transferred to a primary culture dish and supplemented with low glucose Dulbecco's Modified Eagle Medium (DMEM; Life Technologies Pty Ltd., Australia) containing 10% fetal bovine serum (FBS; *In Vitro* Technologies, Australia) and 1% penicillin/streptomycin (P/S; Life Technologies Pty Ltd., Australia) at 37°C in 5% CO_2_. After reaching 80% confluence, cells were passaged and replated in cell culture flasks. Cell characterization for PDLCs and DPCs has been carried out in our previous study [21]. For hypoxic exposure, cells were cultured in a hypoxic chamber flushed with 2% O_2_ and 5% CO_2_, with balance of 93% N_2_ at 37°C [22].

### 2.2. Evaluation of Cell Proliferation

PDLCs and DPCs were cultured in 96-well plates either in normoxia (20% O_2_) or hypoxia (2% O_2_) at an initial density of 4 × 10^3^ cells per well. On days 1, 3, and 7, 20 *μ*L of 3-(4,5-dimethylthiazol-2-yl)-2,5-diphenyltetrazolium bromide (MTT) solution (0.5 mg/mL; Sigma-Aldrich, Australia) was added to each well and incubated at 37°C. The supernatants were removed after 4 h and replaced with 100 *μ*L dimethyl sulfoxide (DMSO) to solubilize the MTT-formazan product. The absorbance was measured at a wavelength of 495 nm with a microplate reader (SpectraMax, Plus 384, Molecular Devices, Inc., USA).

### 2.3. Osteogenic Differentiation

Osteogenic induction was stimulated using growth medium (low glucose DMEM with 10% FBS and 1% P/S) containing 10 mM *β*-glycerophosphate (Sigma-Aldrich, Australia), 50 *μ*M ascorbic acid (Sigma-Aldrich, Australia), and 100 nM dexamethasone (Sigma-Aldrich, Australia). After two weeks of culture in normoxia (20% O_2_) or hypoxia (2% O_2_), the osteogenically inducted cells were fixed with methanal and the presence of calcium nodules was assessed by Alizarin red S staining.

### 2.4. Chondrogenic Differentiation

PDLCs and DPCs were chondrogenically differentiated by culturing in high cell density through pellet culture (2 × 10^5^ cells per pellet) in 500 *μ*L chondrogenic differentiation medium. Serum-free chondrogenic differentiation medium consisted of high glucose DMEM supplemented with 10 ng/mL of transforming growth factor-*β*3 (TGF-*β*3; R&D Systems, Australia), 10 nM dexamethasone, 50 mg/mL of ascorbic acid, 10 mg/mL of sodium pyruvate (Sigma-Aldrich, Australia), 10 mg/mL of proline (Sigma-Aldrich, Australia), and an insulin-transferrin-selenium supplement. Pellets were allowed to differentiate under 3-dimensional conditions in 15 mL centrifuge tubes at 2% or 20% O_2_ tension. After 3 weeks of chondrogenic differentiation, the pellets were fixed with 4% paraformaldehyde (PFA) and embedded in paraffin. Blocks were cut into 5 *μ*m sections and GAG was detected using Safranin O staining.

### 2.5. Adipogenic Differentiation

Adipogenic differentiation was induced by replacing medium with high glucose DMEM containing 10% FBS, 1% P/S, 0.5 mM isobutylmethylxanthine (Sigma-Aldrich, Australia), 200 *μ*M indomethacin (Sigma-Aldrich, Australia), 1 *μ*M dexamethasone, and 10 *μ*g/mL insulin (Sigma-Aldrich, Australia). After completion of 3 cycles of adipogenic induction [23], cells were kept in adipogenic maintenance medium (10 *μ*g/mL insulin in high glucose DMEM with 10% FBS and 1% P/S) for three weeks, with change of medium every 3 days. After this, cells were washed with PBS, fixed with 4% PFA, and stained with Oil red O to detect the lipid droplets within the differentiated cells cultured in normoxia and hypoxia.

### 2.6. qRT-PCR

Total RNA was extracted from PDLCs and DPCs after culturing in normoxia and hypoxia for 1 day and 1 week with TRIzol Reagent (Ambion, Life Technologies Pty Ltd., Australia). Complementary DNA was synthesized using Superscript III reverse transcriptase (Invitrogen Pty Ltd., Australia) from 1 *μ*g total RNA following the manufacturer's instructions. qRT-PCR was performed on an ABI Prism 7300 Real-Time PCR system (Applied Biosystems, Australia) with SYBR Green detection reagent. The mRNA expression of Oct-4, Sox2, c-Myc, runt-related transcription factor 2 (Runx2), SRY-box 9 (Sox9), and peroxisome proliferator-activated receptor *γ*2 (PPAR*γ*2) was assayed and normalized against glyceraldehyde 3-phosphate dehydrogenase (GAPDH) housekeeping gene. All experiments were repeated at least three times for each sample. For the calculation of fold change, ΔΔCt method was applied to compare mRNA expression between cells cultured in normoxia and hypoxia.

### 2.7. Western Blotting

Total protein was harvested by lysing the cells in a lysis buffer containing a protease inhibitor cocktail (Roche Products Pty. Ltd., Australia). The protein concentration was determined by a bicinchoninic acid (BCA) protein assay kit (Sigma-Aldrich, Australia). 10 *μ*g of protein from each sample was separated on SDS-PAGE gels and transferred onto a nitrocellulose membrane (Pall Corporation, USA). The membranes were incubated with primary antibodies against HIF-1*α* (1 : 1000, mouse anti-human, Novus Biologicals, Australia), Oct-4 (1 : 1000, mouse anti-human, Santa Cruz, Australia), Sox2 (1 : 1000, goat anti-human, Santa Cruz, Australia), c-Myc (1 : 1000, mouse anti-human, Santa Cruz, Australia), and *α*-Tubulin (1 : 5000, rabbit anti-human, Abcam, Australia) overnight at 4°C. The membranes were washed three times in TBST buffer and then incubated with a corresponding secondary antibody at 1 : 2000 dilutions for 1 h. The protein bands were visualized using the ECL Plus Western Blotting Detection Reagents (Thermo Fisher Scientific, Australia) and exposed on X-ray film (Fujifilm, Australia). Band intensities of HIF-1*α* were quantified by scanning densitometry and analysed using ImageJ software.

### 2.8. Statistical Analysis

Analysis was performed using SPSS software (SPSS Inc., Chicago, IL, USA). All the data were presented as means ± standard deviation (SD) and analysed using the nonparametric Wilcoxon test to distinguish the differences between the two groups (normoxic culture and hypoxic culture). A *P* value < 0.05 was considered statistically significant.

## 3. Results

### 3.1. Confirmation of Cellular Hypoxia

**Figure 1:**
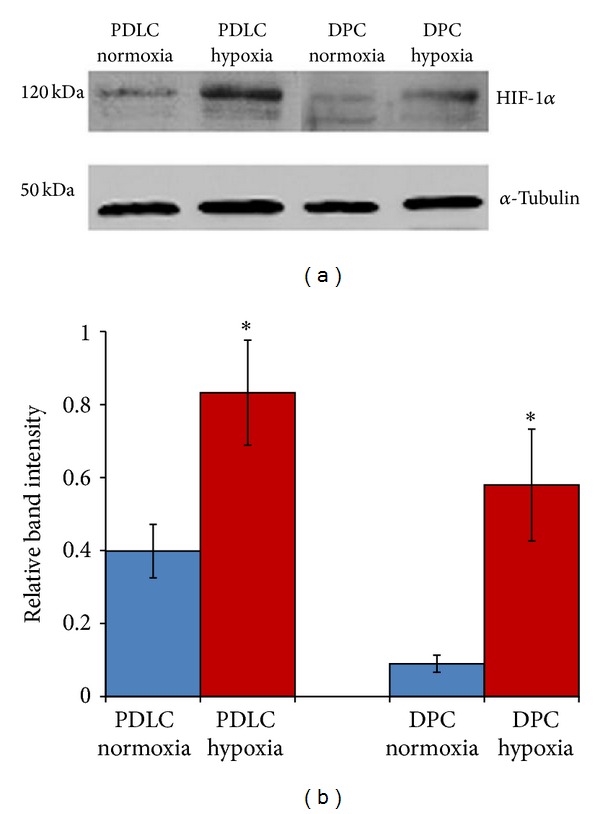
Confirmation of cellular hypoxia. (a) Western blotting analysis revealed degradation of HIF-1*α* protein in PDLCs and DPCs cultured in normoxia and stable HIF-1*α* protein expression in the hypoxic samples. (b) Quantification of the western blots (*P < 0.05).

To confirm that PDLCs and DPCs metabolically respond to hypoxic culture conditions, we assessed whether HIF-1*α* was activated in the cells exposed to 2% O_2_. As demonstrated in Figure 1(a), HIF-1*α* was detected in both PDLCs and DPCs after exposure to hypoxia. Quantification of the western blots showed a remarkable degradation of HIF-1*α* when PDLCs and DPCs were cultured in normoxia (Figure 1(b)).

### 3.2. Effects of Hypoxia on Cell Viability

**Figure 2:**
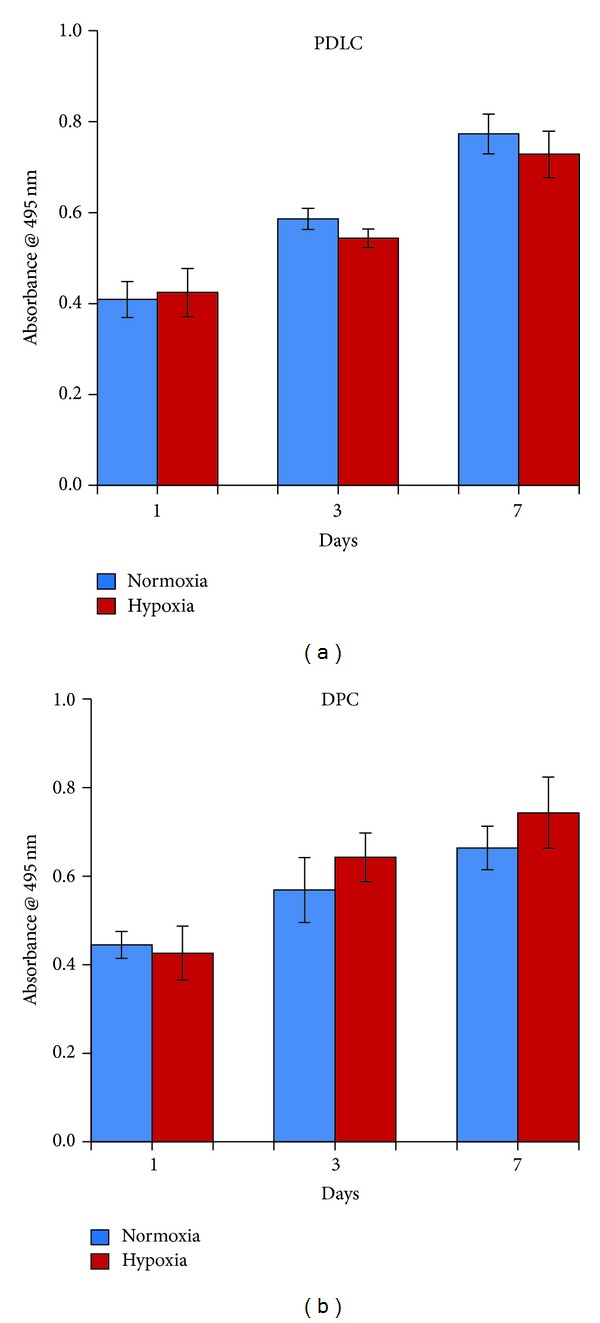
Cell proliferation of PDLCs and DPCs. No significant difference was observed in PDLCs (a) and DPCs (b) cultured in normoxia and hypoxia (*P* > 0.05).

There was no significant difference in the proliferation rate of PDLCs and DPCs cultured under the normoxic and hypoxic conditions (Figures 2(a) and 2(b)). However, there was a slight upward trend of cell growth in a time-dependent manner in PDLCs and DPCs cultured in hypoxia.

### 3.3. Effect of Hypoxia on Stemness Maintenance

**Figure 3:**
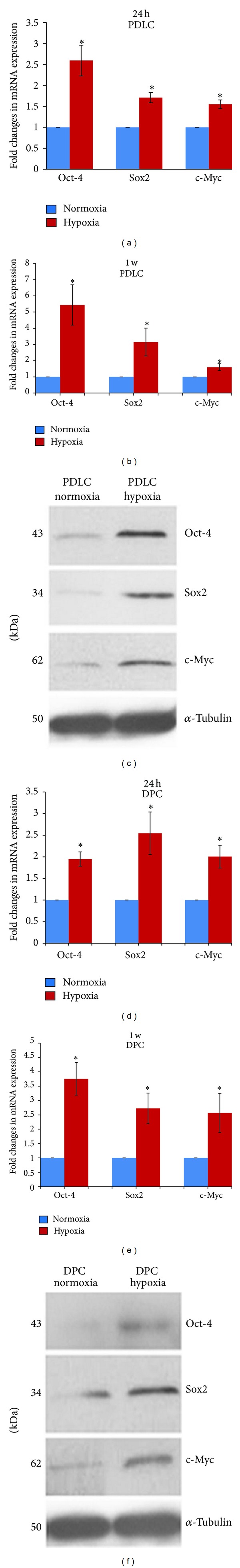
Effect of hypoxia on the mRNA expression levels of Oct-4, Sox2, and c-Myc in PDLCs and DPCs. The mRNA expressions of Oct-4, Sox2, and c-Myc in PDLCs significantly increased after exposure to 2% O_2_ for 24 h (a) till 7 days (b) (*P < 0.05). Western blotting analysis further confirmed the result in protein level (c). The expressions of Oct-4, Sox2, and c-Myc in DPCs cultured in hypoxia showed a similar pattern ((d)–(f)) (*P < 0.05).

Hypoxia led to an increased level of mRNA expression of Oct-4, Sox2, and c-Myc in PDLCs and DPCs. The expression of these pluripotency markers was elevated after the cells were cultured under hypoxic conditions for 24 h (Figures 3(a) and 3(d)) and maintained a statistically significant increase on day 7 (Figures 3(b) and 3(e)). The protein expression of these markers (Oct-4, Sox2, and c-Myc) showed a similar trend of upregulation in hypoxic environment (Figures 3(c) and 3(f)).

### 3.4. Hypoxia Enhanced Differentiation Potential of PDLCs and DPCs

**Figure 4:**
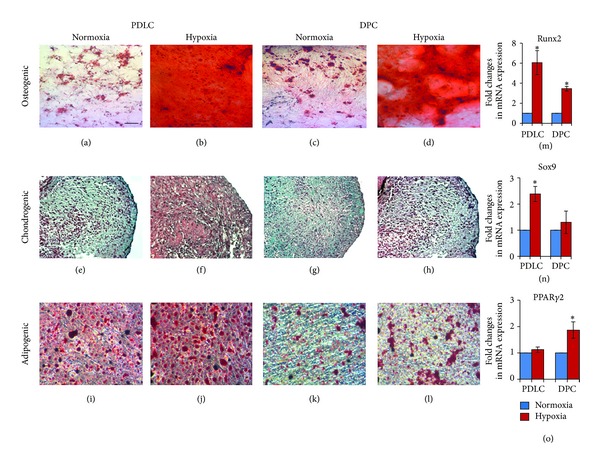
Effect of hypoxia on the osteogenic, chondrogenic, and adipogenic differentiation of PDLCs and DPCs. PDLCs and DPCs that have been osteogenically induced under hypoxic conditions ((b) and (d)) display strong Alizarin red S staining compared to those cultured in normoxia ((a) and (c)). Chondrogenically differentiated PDLCs and DPCs ((f) and (h)) showed higher Safranin O staining intensity when cultured in hypoxia. A larger number of lipid droplets were observed in PDLCs and DPCs after exposure to hypoxia ((j) and (l)) than those cultured in normoxia ((i) and (k)). Bar = 50 *μ*m. The mRNA expression of Runx2, Sox9, and PPAR*γ*2 in PDLCs and DPCs increased after differentiation towards different lineages under hypoxic conditions ((m), (n), and (o)).

Assay of the differentiation potential of PDLCs and DPCs towards osteo-, chondro-, and adipogenic cell lineages showed considerable variation in different culture microenvironments. More calcium deposits were observed in PDLCs and DPCs after 2 weeks of osteogenic induction in hypoxia (Figures 4(b) and 4(d)) than in normoxia (Figures 4(a) and 4(c)) as revealed by Alizarin red S staining. Under chondrogenic induction, PDLCs and DPCs cultured in hypoxia showed higher staining intensity of proteoglycan-rich extracellular matrix deposition (Figures 4(f) and 4(h)). With regard to adipogenic differentiation, PDLCs and DPCs showed a larger number of clusters of lipid droplets after exposure to hypoxia (Figures 4(j) and 4(l)) than those cultured in normoxia (Figures 4(i) and 4(k)). Our qRT-PCR results showed a significant increase in the mRNA expression of Runx2 after PDLCs and DPCs were inducted towards osteogenic lineage in hypoxia (Figure 4(m)). The hypoxic treatment also led to the enhanced mRNA expression of Sox9 in PDLCs (Figure 4(n)) and PPAR*γ*2 in DPCs (Figure 4(o)).

## 4. Discussion

To prevent immunological rejection and unexpected infectious diseases in regenerative therapy, one of the solutions is to use the patient's own cells. It is necessary to maximize the pluripotency of the donor cells when they are maintained in culture prior to their differentiation into a specific target lineage [24]. However, the therapeutic potential of PDLCs and DPCs is hindered by an incomplete understanding of *in vitro* culture conditions that can maintain their stemness and multipotent potential during expansion. Most of the currently identified regulators of stem cell fate are transcription factors and cell cycle regulators such as Oct-4, Sox2, and c-Myc as well as the downstream signalling pathways [25]. A recent study confirms that low oxygen level can activate Oct-4 and may act as a key inducer of a dynamic state of stemness in cancer cells [26]. Oxygen is critical for cellular energy production and metabolism. Previous studies have shown that hypoxia may induce the expression of HIF-1*α*, which regulates the expression of different target genes affecting the embryonic development [27], cell proliferation [28], differentiation [29], and apoptosis [30]. Recent studies have also revealed that hypoxia is related to the maintenance of undifferentiated state of stem cells in the neural crest and central nervous system [31].

In this study, PDLCs and DPCs were cultured under hypoxic conditions in 2% O_2_, compared to the normal culture conditions in 20% O_2_. Our results indicated that hypoxia did not negatively affect the proliferation of PDLCs and DPCs. Enhanced expression of Oct-4, Sox2, and c-Myc in PDLCs and DPCs cultured in hypoxia was observed, suggesting that low O_2_ microenvironment may be necessary for triggering the expression of these stem cell markers to maintain the stem cell properties of adult PDLCs and DPCs, although the molecular signalling pathways connecting hypoxia and stemness are yet to be elucidated.

It was also shown in our study that *in vitro* hypoxic culture enhanced differentiation potential of PDLCs and DPCs as evidenced by the significantly greater amount of calcified nodules, GAG deposition, and lipid droplets formation. Furthermore, the mRNA levels of Runx2, Sox9, and PPAR*γ*2 increased after the differentiation of PDLCs and DPCs towards different lineages. These findings suggest that 2% O_2_ hypoxic treatment may promote differentiation potential of PDLCs and DPCs. Even though the mechanism by which hypoxia influences the differentiation capacity of PDLCs and DPCs is not clearly understood, it could be speculated that HIF-1*α* is activated in PDLCs and DPCs after exposure to hypoxia and then induces cell signalling pathways such as Wnt, Notch, and Sonic hedgehog (Shh), which help maintain the cell stemness and enhance the differentiation capacity [32, 33]. Further studies need to be conducted to clarify the molecular mechanisms behind these hypoxia-related phenomena.

## 5. Conclusion

The present study demonstrates that hypoxic microenvironment can maintain proliferation capacity, enhance pluripotency marker expression, and promote differentiation potential of PDLCs and DPCs. Our results indicate that effective isolation and expansion of PDLCs and DPCs under the hypoxic conditions may be a very important technique for autologous cell-based therapy. Further investigation will be performed to reveal the mechanism of hypoxia in maintaining stemness and promoting differentiation potential of PDLCs and DPCs.
